# The Standardized Letter of Evaluation Narrative: Differences in Language Use by Gender

**DOI:** 10.5811/westjem.2019.9.44307

**Published:** 2019-10-17

**Authors:** Danielle T. Miller, Danielle M. McCarthy, Abra L. Fant, Simiao Li-Sauerwine, Aimee Ali, Amy V. Kontrick

**Affiliations:** * Stanford University School of Medicine, Department of Emergency Medicine, Palo Alto, California; † Northwestern University Feinberg School of Medicine. Department of Emergency Medicine, Chicago, Illinois; ‡ Ohio State University College of Medicine, Department of Emergency Medicine, Columbus, Ohio

## Abstract

**Introduction:**

Prior research demonstrates gender differences in language used in letters of recommendation. The emergency medicine (EM) Standardized Letter of Evaluation (SLOE) format limits word count and provides detailed instructions for writers. The objective of this study is to examine differences in language used to describe men and women applicants within the SLOE narrative.

**Methods:**

All applicants to a four-year academic EM residency program within a single application year with a first rotation SLOE available were included in the sample. We used the Linguistic Inquiry and Word Count (LIWC) program to analyze word frequency within 16 categories. Descriptive statistics, chi-squared, and t-tests were used to describe the sample; gender differences in word frequency were tested for using Mann-Whitney U tests.

**Results:**

Of 1117 applicants to the residency program, 822 (82%) first-rotation SLOEs were available; 64% were men, and 36% were women. We did not find a difference in baseline characteristics including age (mean 27 years), top 25 schools (22.5%), Alpha Omega Alpha Honor Medical Society rates (13%), and having earned advanced degrees (10%). The median word count per SLOE narrative for men was 171 and for women was 180 (p = 0.15). After adjusting for letter length, word frequency differences between genders were only present in two categories: social words (women: 23 words/letter; men: 21 words/letter, p = 0.02) and ability words (women: 2 words/letter; men: 1 word/letter, p = 0.04). We were unable to detect a statistical difference between men and women applicants in the remaining categories, including words representing communal traits, agentic traits, standout adjectives, grindstone traits, teaching words, and research words.

**Conclusion:**

The small wording differences between genders noted in two categories were statistically significant, but of unclear real-world significance. Future work is planned to evaluate how the SLOE format may contribute to this relative lack of bias compared to other fields and formats.

## INTRODUCTION

Gender disparities exist in academic medicine. Women in academic medicine are less likely to achieve the rank of professor or hold senior leadership positions compared to men, even after adjusting for age, experience, specialty, and research productivity.^1^,^2^ Previous studies in other professional fields have shown that there are differences in language used in describing men and women in letters of recommendation.^3^–^5^ Additional studies have shown that evaluations of women medical students are more likely to describe women as “caring,” “compassionate,” and “empathetic,” in addition to “bright” and “organized,” than male medical students.^6^–^8^ In addition, women are more often portrayed as teachers and students, and less often portrayed as researchers or professionals compared to men.^9^

Within emergency medicine (EM) the letter of recommendation, including both standardized letters and traditional letters, has been cited as one of the top four most important factors in selecting applicants to residency, along with EM rotation grade, interview, and clinical grades.^10^ More specifically, the letter of recommendation has been cited as the most important factor in selecting applicants to interview.^11^ Historically, in EM, letters of recommendation were written without guidelines or restrictions. In 1996, the Council of Residency Directors in Emergency Medicine (CORD) implemented the standardized letter of recommendation (SLOR), which was renamed the standardized letter of evaluation (SLOE) in 2013. The SLOE contains both a quantitative evaluation of an applicant and a narrative portion of 250 words or less.^12^–^14^ The SLOE narrative provides a focused assessment of the non-cognitive attributes of potential residency candidates.^15^

The standardized format and universal instructions make the SLOE a good text sample to study for variation in language by gender. Additionally, while there are several studies analyzing traditional letters of recommendation for language variation between genders, there is a gap in the current literature in analyzing standardized letters of recommendation. Previously, our research team published a study in *Academic Emergency Medicine Education and Training* that showed minimal differences in language use between genders in evaluating 237 SLOEs from applicants invited to interview to a single academic EM residency for the 2015–2016 application cycle.^16^ The small dataset, and potential for a homogeneous sample (as only the SLOEs of applicants invited to interview were included), prompted the current investigation with a goal of confirming or refuting the original results with a larger dataset.

The choice to include all applicants was made with a goal of potentially increasing the variability in the language used within the SLOE (e.g., word frequency in one word category may be equal across genders for the strongest applicants invited to interview, as in our first study, but a gender gap may be unveiled in a larger sample of all applicants). The aim of this study was to compare differences in language within specific word categories to describe men and women applicants in the SLOE narrative for all applicants to a single academic EM residency program for the 2016–2017 application cycle. We secondarily sought to determine whether there was an association between word categories’ differences and invitation to interview, regardless of gender, in order to better contextualize the possible importance of wording differences.

## METHODS

### Study Design

This was a cross-sectional descriptive study employing a linguistic analysis to describe features of the words used in the narrative portion of the SLOE for all applicants during one application cycle. This study was reviewed by the institutional review board at Northwestern University and deemed exempt.

**Population Health Research Capsule**

What do we already know about this issue?

*Prior research demonstrates that there are gender differences in the language used to describe women and men applicants in letters of recommendation*.

What was the research question?

*Within the emergency medicine (EM) SLOE narrative, are there differences in language used to describe women and men applicants*.

What was the major finding of the study?

*Small wording differences exist in SLOE narratives between genders in two of sixteen word categories*.

How does this improve population health?

*The standardized format of the EM SLOE may limit gender bias within the letter of recommendation relative to other fields and formats*.

### Study Setting and Population

Northwestern University McGaw Medical Center EM residency is a four-year, urban, academic residency program with 60 total residents. Applications to the residency program are accepted through the Electronic Residency Application Service (ERAS), which transmits applications, letters of recommendation, medical student performance evaluations, and transcripts to residency programs. Applicants must participate in the National Resident Matching Program (NRMP) to be eligible for selection to the residency.

### Study Protocol

SLOE narratives for all applicants to the residency for the application cycle 2016–2017 were downloaded from ERAS by the program coordinators and converted to Microsoft Word format. We included the narrative portion of the SLOE in analysis. The narrative is limited to 250 words and asks the writer to “Please concisely summarize this applicant’s candidacy including… (1) Areas that will require attention, (2) Any low rankings from the SLOE, and (3) Any relevant noncognitive attributes such as leadership, compassion, positive attitude, professionalism, maturity, self-motivation, likelihood to go above and beyond, altruism, recognition of limits, conscientiousness, etc.”^15^ If applicants submitted more than one SLOE, the SLOE from the first chronological clinical EM rotation was included in analysis. We analyzed first-rotation SLOEs, as opposed to all SLOEs, to provide a uniform evaluation of student performance and limit word differences based on varying experiences in time. Additionally, not every applicant had more than one SLOE. Exclusion criteria included applicants from non-Liaison Committee on Medical Education (LCME) schools, as well as applicants with a first-rotation SLOE that was not available to be downloaded from ERAS. Analysis began after all NRMP decisions had been made and finalized and did not affect an applicant’s invitation to interview or placement on the rank list.

Prior to analysis, each letter was read by two reviewers who screened for “stock” language. These “stock” or standardized sentences were not related to applicant characteristics. They included statements in certain categories such as statements regarding waiving rights to see the letter (“The student has waived his or her right to see this letter”); stock opening statements (“This is a composite letter”); stock closing statements (“Please contact me if you have any questions”); descriptors of the rotation (“The student rotated at a site with 110,000 visits of year…”); descriptors of grade calculation (“We calculate a numerical grade for each of the following 5 areas...”); and descriptors of the letter writer (“As department chair…”). Any letter-writer signatures and titles were deleted prior to analysis to avoid introducing bias. Pronouns were not made pleural (eg, his/her) or deidentified prior to analysis.

### Measures

Measures obtained from the ERAS application for use in describing the sample included the following: age at time of application; gender; Alpha Omega Alpha (AOA) Honor Medical Society designation at the time of application; and advanced degrees. Medical school rank was obtained from the 2016 *US News and World Report* rankings for medical schools in the research category. 17 We did not use class rank as it is not a standardized measure across medical schools.

The analysis approach was the same as that taken in our prior study.^16^ In short, the Linguistic Inquiry and Word Count (LIWC)^18^ is a text analysis dictionary composed of 80 word categories with 4500 words and word stems. We employed the LIWC program in our study to provide word counts and ratios of words per SLOE for each individual SLOE text file. Within the 80 word categories we selected 16 word categories for comparison based on prior research that has evaluated gendered language in professional letters of recommendation.^3^,^4^,^8^ These word categories have also been used in the medical literature.^8^ In other studies within the medical literature that do not use the LIWC categories, similar words and word categories overlapped with the LIWC word categories selected.^6^,^7^

These 16 categories included nine taken from the default LIWC 2015 dictionary: positive emotion (eg, nice); negative emotion (eg, nasty); social words (eg, friend); cognitive processes (eg, knowledge); affiliation words (eg, social); achievement words (eg, success); power words (eg, superior); reward words (eg, benefit); and risk words (eg, doubt). The remaining seven categories were “user-defined dictionaries,” which have been previously generated for studies of gender and letters of recommendation.^3^–^5^, ^9^, ^22^ These categories include “grindstone” traits (eg, diligent); ability traits (eg, talented); standout adjectives (eg, exceptional); research terms (eg, project); teaching terms (eg, teach); communal traits (eg, kind, caring); and agentic characteristics (eg, ambitious, confident). The LIWC software reports word counts and ratios of words per SLOE for all 16 word categories.

To validate the LIWC tool and dictionaries, independent judges rated hundreds of text samples, and then their ratings were compared to computerized LIWC ratings of the same text.^19^–^21^ The communal and agentic word dictionaries were validated by Madera and colleagues by having independent judges rate the letters as a whole on a scale of 1–9 for the “degree to which the applicant is described as communal/agentic” and subsequently evaluated for correlation of those scores to the LIWC word-count frequencies.^3^ The additional five word dictionaries have not been externally validated.

### Data Analysis

We used descriptive statistics to report the applicants’ characteristics and assessed for differences in baseline characteristics by gender using t-tests and chi-squared tests, as appropriate. Median word counts for the identified 16 categories of interest (nine LIWC default categories, seven user-defined dictionaries) were reported. For the primary outcome of interest, we assessed differences by gender in word counts after adjusting for letter length using Mann-Whitney U tests. In secondary analysis, the analyses were repeated for differences in word categories by invitation to interview. We used multivariable logistic regression to identify word categories associated with receiving an invitation to interview. Covariates in this model were selected via a predetermined inclusion threshold of α = 0.10. We performed all analyses using Stata 13.1 (College Station, TX).

Additionally, for any of the seven user-defined word categories in which a difference was noted, a further analysis was conducted evaluating the use of each individual word in the dictionary to assess if the difference for the category was driven by the use of a single word (eg, talented, bright), or by the use of multiple descriptors within the category. For this analysis, the proportion of SLOEs with each word included was compared by gender using Fisher’s exact test. This analysis was not conducted for any differences in the LIWC defined categories due to the size of the word dictionaries (eg, >700 social words in LIWC dictionary vs 15–40 words in user-defined dictionaries).

## RESULTS

**Figure 1:**
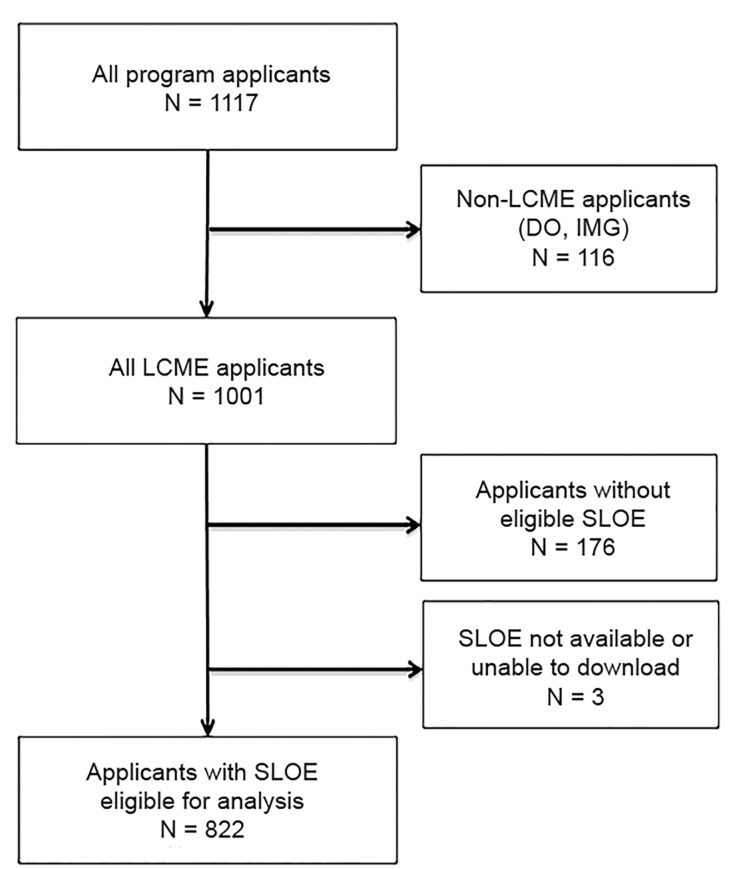
Selection of Standardized Letter of Evaluation (SLOE) for inclusion in analysis. *DO*, doctor of osteopathic medicine; *IMG*, international medicine graduate; *LCME*, Liaison Committee on Medical Education.

There were 1117 applicants to the residency in the single application cycle of study (2016–2017) of whom 1001 were graduates from LCME schools (Figure 1). We included in this study the 822 applicants (82%) who had a first-rotation SLOE available for analysis. Of these, 64% of applicants were men, and 36% were women. Comparing men and women applicants, we found no differences detected between genders for baseline characteristics including age (mean 27 years); top 25 schools (22.5%); AOA rates (13%); and having earned advanced degrees (10%) (Table 1).

The median word count per SLOE narrative for men was 171 (interquartile range [IQR] 127–224) and for women was 180 (IQR 133–225), which was not statistically different (p = 0.15). Within the 16 word categories investigated, after adjusting for letter length, word frequency differences between genders were only present in two categories: social words (women: 23 words/letter; men: 21 words/letter, p = 0.02) and ability words (women: 2 words/letter; men: 1 word/letter, p = 0.04) (Table 2).

The remaining categories, including words representing communal or agentic traits, standout adjectives, grindstone traits, and teaching and research words were also not statistically different between men and women applicants. Among ability words, there were no significant differences in the number of SLOEs for men or women using specific words within the ability word dictionary (see Appendix 1).

In a secondary analysis comparing applicants invited to interview and those not invited to interview, regardless of gender, invited applicants had slightly longer SLOEs (median 17 words longer) with significantly more standout, ability, power, and research words. The differences in all word categories were small (Table 3).

Notably, invited applicants had fewer reward words than non-invited applicants. In adjusted analysis, letters with standout words were associated with the highest odds of receiving a request to interview (OR [odds ratio[ 1.15, 95% confidence interval [CI],1.05–1.26), and letters with reward words had the lowest odds of receiving a request to interview (OR 0.89, 95% CI, 0.82–0.95). Other word categories were no longer significantly associated with higher or lower odds of receiving an interview after adjustment.

## DISCUSSION

This analysis found small but quantifiable differences in word frequency between genders in the language used in the SLOE. In this study, differences between genders were present in two categories: social words and ability words, with women having higher word frequency in both categories. Our prior investigation found differences of similar magnitude (eg, one word differences per letter) in affiliation words and ability words, with letters for women applicants having higher word frequency in both categories. For both studies, the differences in word frequency were statistically significant, but it is difficult to comment or draw conclusions about the significance of these small wording differences on application or educational outcomes. What is perhaps more notable than the presence of differences in two categories is the lack of difference in the remaining 14 categories.

When looking specifically at the categories that had gender differences, our finding of ability words being used to describe women applicants more frequently than men applicants is in contrast to previous studies, while our other research finding, that women are more frequently described with social words than men, is in alignment with previous studies. In the medical literature, letters of recommendation for men applying for faculty positions contain more ability attributes such as standout adjectives and research descriptors than letters for women,^9^ and letters for women in medical school applying for residency positions are more frequently described by non-ability attributes such as being caring, compassionate, empathetic, bright, and organized.^6^

Looking specifically at ability words, this word category had statistically significant differences in both this investigation and our prior study, with ability words occurring more frequently for women than men. Ability words include descriptors such as talented, skilled, brilliant, proficient, adept, intelligent, and competent. This consistency of findings between the two samples suggests that letter writers employ multiple descriptors within the ability category to convey proficiency of women applicants. However, the reason for this difference is unclear. Notably, the word “bright” is one of the ability words for which there was no gender difference found, counter to findings from prior research wherein women applicants were more often described as bright.^6^,^18^ While the descriptor “bright” is often considered a compliment, it has also been suggested that its use “subtly undermines the recipient of the praise in ways that pertain to youth and, often, gender” stemming from its association with the phrase “bright young thing.” 23

The finding that women were more frequently described with social words (two words more frequently than men) aligns with previous studies of letters of recommendations. Studies in letters of recommendation for psychology and chemistry faculty positions have shown that women are often described as communal (eg, warm, kind), while men are described as agentic (eg, dominant, confident) and have more standout adjectives (eg, exceptional).^3^,^9^ Other studies have found women to be described as more communicative.^6^

We employed a secondary analysis with respect to the invitation to interview to determine if small differences in word categories were associated with invitation to interview. The adjusted analysis showed an association between more standout words and invitation to interview; however, this analysis did not account for other factors that may influence invitations to interview (eg, school rank, grades). Although these findings represent an association and not causation, they help to contextualize the potential importance of small differences in word use, although this is not conclusive. Notably, neither social words nor ability words (the categories in which there were gender differences) influenced the choice to interview, and there was an equitable frequency of standout words between genders.

Despite the small word differences in the categories of social and ability words, we did not find a difference in the 14 other word categories queried. There are several possible explanations for this lack of a finding. It is possible that the sample was underpowered to detect small wording differences in the 14 word categories. Another explanation is that the SLOE format itself may be driving the lack of a difference. The short word format of the SLOE (limiting to 250 words) and specific, detailed instructions as noted above may reduce bias. Other explanations include the increasing use of group authorship, which may introduce less bias than individual authorship. In 2012, a sampling of three EM residencies calculated that 34.9% of SLORs were created by groups.^24^ In 2014, 60% of EM program directors (PD) participated in group SLORs, 85.3% of departments provided a group SLOR, and 84.7% of PDs preferred a group SLOR.^11^ Although the sample size and lack of a standard comparator (eg, SLOE and full-length letter on each candidate from the same author) limit the ability to determine why we did not find a difference for the majority of word categories, we hypothesize that it is related to the format and hope to further support that hypothesis through future work examining paired SLOE and full-length letters for candidates.

A recently published study by Friedman and colleagues in the otolaryngology literature has been the only study, in addition to our own, to our knowledge that evaluates a standardized letter for gender bias. In this 2017 study, the SLOR and more traditional NLOR (Narrative Letter of Recommendation) in otolaryngology residency applications were compared by gender, concluding that the SLOR format reduced bias compared to the traditional NLOR format. Although in both letter formats some differences persisted (eg, women more frequently described as “team players”), the SLOR format resulted in less frequent mention of women’s appearance and more frequent descriptions of women as “bright.”^22^ Although their analysis strategy differed from the one we used in this study, their findings parallel ours in that there are minimal differences by gender in a restricted letter format and highlight the need for further study of the how the question stem and word limitations may be intentionally built to minimize bias.

Lastly, of note, our study focused specifically on differences in language use in the SLOE. This study does not evaluate the presence or absence of gender bias in the quantitative aspects of the SLOE, nor does our multivariable model include other factors that would influence the invitation to interview such as rotation grades, test scores, school rank, or AOA status. Such analyses were beyond the scope of our study, which was focused on the SLOE narrative itself. Other studies have evaluated this but have not evaluated the narrative portion of the SLOE.^25^

Additionally, there remain many other forms of evaluation, numerical and narrative, in medical training, in addition to the SLOE that have analyzed gender bias. Recent studies have suggested that bias persists in other forms of evaluation. Specifically, Dayal and colleagues’ recent publication notes lower scores for women residents in EM Milestones ratings compared to male peers as they progress through residency.^26^ Evaluations of narrative comments from shift evaluations are another area of interest, of which we are aware of two current investigations underway in EM programs. Additionally, a study of evaluations of medical faculty by physician trainees by Heath and colleagues also showed gender disparities.^27^ As this body of literature continues to grow and interventions are developed to minimize bias in all narrative performance evaluations, we believe it will be important to think carefully about the question stems and response length allowed. Unfortunately, limiting space may also limit the room for positive evaluation and strings of praising adjectives.^22^ However, while implicit bias exists, employing limits in response format may rein in the manifestation of implicit bias by focusing the writer.

## LIMITATIONS

This was a single center study; only SLOE narratives from applicants who applied to interview at a single, academic EM residency program were included in analysis, and applicants from non-LCME schools were excluded, limiting generalizability. The man to woman applicant ratio in this study reflects the national trend for the 2017 match, which may contribute to generalizability. 28 ERAS does not allow an individual program to access SLOEs for applicants who have not selected that program; therefore, a full national sample of all applicants in a single year to ERAS was not feasible.

Our analysis used the LIWC linguistic software and focused on individual words. Other approaches, such as qualitative content analysis or focusing on phrases (eg, leadership potential) or searching for specific words (eg, bright) as was done by Friedman and colleagues in the study discussed above may have yielded different findings. Additionally, the LIWC contains pre-established word lists. While these lists have been used in medical literature,^8^ it is possible that there may be a set of words for EM that is more applicable.

Our analysis used word frequency as a measurement of biased language and did not evaluate context of the words in the letters, limiting the study. Words in different contexts can have different meaning. For instance, the word “aggressive” can have both a positive or negative connotation based on context when describing and applicant as “aggressive in picking up patients” vs “aggressive with consultants.” A qualitative analysis of the SLOEs would better delineate the context of word phrases and provide a more in-depth analysis.

Although it is a limitation that we did not evaluate word context, word frequency software applied to a large sample gives generalizability that a small qualitative analysis may not be able to achieve. In these rare instances of context misinterpretation for positive and negative emotion categories (ie, as stated in the previous example with the word “aggressive,” which would be interpreted in the software as a negative emotion word category), this may be of little overall consequence as there is such a large margin between median positive vs negative words within these word categories (median 10 vs median 1, Table 2). Additionally, the subtle differences between word phrases such as “we strongly recommend this student” vs “we will be recruiting this student” would not be picked up by the software.

This was an exploratory study and as such was not powered to a specific outcome; however, we estimated that with our sample size of 822 (allocation 1.5 male/female) that we would have 80% power to detect a difference of 0.2 mean words within a single word category with a 5% type I error (based on estimated baseline word frequency per category of three words). Additionally, it is possible that the sample was underpowered to detect small wording differences among the 16 word categories, which could represent a type II error. The analysis for differences in 16 categories raises the question of the multiple comparisons problem.

We did not replicate the findings of our previous study with regard to differences in the same word categories, further adding to this concern. However, we are equally interested in the lack of a difference as we are in detecting differences. Although negative findings are often highlighted less than positive ones, this analysis did not find a difference in the majority of word categories (a finding that is similar to the prior study). Finally, as the majority of letters do not denote letter-writer gender and most were composed by a group of authors, this group composition did not allow for any evaluation of the relationships between author gender and applicant gender with respect to language used in the SLOE.

## CONCLUSION

This study expanded upon our prior work by employing a larger dataset–all applicants to a single residency program–rather than only the highest achieving applicants invited to interview. Within this larger study population, minimal differences were detected in word frequency between genders for 16 categories of words. The wording differences noted in two categories were statistically significant, with one to two word differences between genders. Future work will evaluate how the SLOE format may contribute to this relative lack of bias compared to other fields and formats, including a comparison of the SLOE and traditional letters of recommendation submitted for individual EM residency applicants.

## Supplementary Information



## Figures and Tables

**Table 1:** Applicant information Standardized Letter of Evaluation.

Variable	Total n = 822 n (%)	Male n = 526 n (%)	Female n = 296 n (%)	P-value
Age, mean (SD)	27 (2.9)	27 (3.0)	27 (2.8)	0.60
Top 25 Ranked Med School	185 (22.5%)	122 (23.2%)	63 (21.3%)	0.53
AOA	104 (12.7%)	68 (13%)	36 (12.2%)	0.31
Advanced Degree	82 (10%)	55 (10.5%)	27 (9.1%)	0.54

*SD*, standard deviation; *AOA*, Alpha Omega Alpha.

**Table 2:** Select Linguistic Inquiry and Word Count output variables and word categories of the Standardized Letter of Evaluation, comparing male and female applicants.

Variable	Total n = 822 median (IQR)	Male n = 526 median (IQR)	Female n = 296 median (IQR)	P-value
Word count	173 (129–224)	171 (127–224)	180 (133–225)	0.15
Words per sentence	15 (13–18)	15 (13–18)	15 (13–18)	0.17
Positive emotion	10 (8–14)	10 (8–13)	11 (8–14)	0.26
Negative emotion	1 (0–2)	1 (0–2)	1 (0–2)	0.77
Social	21 (16–28)	21 (15–27)	23 (17–28)	0.02
Cognitive processes	14 (9–19)	13 (9–18)	14 (10–19)	0.12
Affiliation	4 (3–6)	4 (2–6)	4 (3–7)	0.38
Achievement	8 (6–11)	8 (6–11)	8 (6–11)	0.07
Power	6 (4–8)	6 (4–8)	6 (4–8)	0.82
Reward	4 (3–6)	4 (3–6)	4 (3–6)	0.42
Risk	0 (0–1)	0 (0–1)	0 (0–1)	0.50
Standout	1 (0–2)	1 (0–2)	1 (0–2)	0.17
Ability	1 (1–3)	1 (0–3)	2 (1–3)	0.04
Grindstone	2 (1–3)	2 (1–3)	2 (1–3)	0.55
Teaching	2 (1–4)	2 (1–4)	2 (1–4)	0.27
Research	0 (0–1)	1 (0–1)	0 (0–1)	0.88
Communal	1 (0–2)	1 (0–2)	1 (0–2)	0.36
Agency	1 (1–2)	1 (0–2)	2 (1–3)	0.08

*IQR*, interquartile range.

**Table 3:** Select Linguistic Inquiry and Word Count output variables and word categories of the Standardized Letter of Evaluation, comparing applicants invited to interview and applicants not invited to interview.

Variable	Total n = 822 median (IQR)	Invited n = 202 median (IQR)	Not invited n = 620 median (IQR)	P-value
Word count	173 (129–224)	186 (135–228)	169 (127–223)	0.03
Words per sentence	15 (13–18)	16 (14–18)	15 (13–18)	0.42
Positive emotion	10 (8–14)	10 (8–13)	10 (8–14)	0.07
Negative emotion	1 (0–2)	1 (0–1)	1 (0–2)	0.89
Social	21 (16–28)	23 (17–28)	21 (15–27)	0.34
Cognitive processes	14 (9–19)	15 (10–20)	13 (9–18)	0.61
Affiliation	4 (3–6)	5 (3–7)	4 (2–6)	0.21
Achievement	8 (6–11)	9 (6–11)	8 (6–11)	0.75
Power	6 (4–8)	6 (4–9)	6 (3–8)	0.02
Reward	4 (3–6)	4 (2–6)	4 (3–6)	0.001
Risk	0 (0–1)	0 (0–1)	0 (0–1)	0.96
Standout	1 (0–2)	2 (1–3)	1 (0–2)	<0.0001
Ability	1 (1–3)	2 (1–3)	1 (0–2)	0.005
Grindstone	2 (1–3)	2 (1–4)	2 (1–3)	0.62
Teaching	2 (1–4)	2 (1–4)	2 (1–4)	0.41
Research	0 (0–1)	1 (0–2)	0 (0–1)	0.03
Communal	1 (0–2)	1 (0–2)	1 (0–2)	0.47
Agency	1 (1–2)	2 (1–3)	1 (0–2)	0.46

*IQR*, interquartile range.
