# Supplementary material for: The Standardized Letter of Evaluation Narrative: Differences in Language Use by Gender
Source: West J Emerg Med. 2019 Oct 17;20(6):948–56. doi: 10.5811/westjem.2019.9.44307 (PMC6860384; doi:10.5811/westjem.2019.9.44307)
Supplement: Supplementary file 1 [file wjem-20-948-s001.docx]

**Appendix 1:**

Table 4: Ability word frequency in Standardized Letter of Evaluation (SLOE) for men and women applicants

| Word | % of male letters using word at least once | % female letters using word at least once | p-value |
| --- | --- | --- | --- |
| Talent | 2.5 | 2.4 | 0.56 |
| Intelligent | 11.6 | 9.5 | 0.20 |
| Smart | 4.4 | 7.2 | 0.07 |
| Skill | 46.0 | 47.0 | 0.43 |
| Ability | 18.1 | 21.0 | 0.18 |
| Genius | 0 | 0 | - |
| Brilliant | 0 | 0.7 | 0.13 |
| Bright | 7.4 | 9.8 | 0.15 |
| Brain | 0.2 | 0.3 | 0.59 |
| Aptitude | 1.3 | 1.7 | 0.45 |
| Gift | 0.8 | 0.3 | 0.41 |
| Capacity | 0.6 | 0.7 | 0.59 |
| Propensity | 0 | 0 | - |
| Innate | 0.4 | 0.7 | 0.46 |
| Flair | 0 | 0 |  |
| Knack | 0.4 | 0.3 | 0.71 |
| Clever | 0 | 0 | - |
| Expert | 0 | 0 | - |
| Proficient | 1.0 | 0 | 0.11 |
| Capable | 3.6 | 2.7 | 0.31 |
| Adept | 2.3 | 2.0 | 0.51 |
| Able | 22.6 | 27.7 | 0.6 |
| Competent | 2.1 | 2.4 | 0.49 |
| Natural | 3.6 | 4.4 | 0.35 |
| Inherent | 0 | 0.7 | 0.13 |
| Instinct | 1.7 | 2.0 | 0.47 |
| Adroit | 0 | 0 | - |
| Creative | 0.2 | 0 | 0.64 |
| Insight | 3.4 | 6.1 | 0.06 |
| Analytical | 0.4 | 0.3 | 0.71 |
